# Complete genome sequence of polyhydroxyalkanoates and carotenoid-producing *Paracoccus marcusii* strain LL1 isolated from Lonar Lake, India

**DOI:** 10.1128/mra.00011-25

**Published:** 2025-07-28

**Authors:** Rhudith B. Cabulong, Aderonke Odunayo Adigun, Samuel Imisi Awala, Krittayapong Jantharadej, Shailesh S. Sawant, Beom Soo Kim

**Affiliations:** 1 Department of Chemical Engineering, Chungbuk National University 34933, Cheongju, Chungbuk, Republic of Korea; 2 Department of Biological Sciences and Biotechnology, Chungbuk National University 34933, Cheongju, Chungbuk, Republic of Korea; 3 Department of Biological Sciences, University of Calgary 2129, Calgary, Alberta, Canada; Indiana University, Bloomington, Indiana, USA

**Keywords:** *Paracoccus marcusii*, carotenoid, PHA, polyhydroxyalkanoates

## Abstract

Microorganisms that co-produce high-value bioproducts, such as polyhydroxyalkanoates (PHA) and carotenoids, are of great industrial interest. Here, we report the complete genome sequence of *Paracoccus marcusii* strain LL1, a bacterium isolated from Lonar Lake, India, that co-produces both PHA and carotenoids.

## ANNOUNCEMENT

*Paracoccus* species can metabolize a wide range of organic compounds and play crucial roles in nitrogen cycling via denitrification (1, 2). Specific *Paracoccus* strains can degrade pollutants and produce useful compounds like polyhydroxyalkanoates (PHA) and carotenoids (2–4). Here, we report the complete genome of *Paracoccus marcusii* strain LL1, which produces PHA and a vivid orange pigment, possibly carotenoids. This strain was isolated from the waters of Lonar Lake, Buldhana district, Maharashtra, India (19°59’N and 76°31’E), spread on minimal salt agar with corn stover hydrolysate at 30°C (4).

*P. marcusii* strain LL1 cells were cultured from −80°C stock in Luria-Bertani broth at 30°C until the exponential phase. High molecular weight genomic DNA (gDNA) was extracted using the i-genomic BYF DNA Extraction Mini Kit (IntronBio, Korea) and sent to Macrogen (Seoul, Korea) for sequencing on PacBio RS II (Pacific Biosciences, Menlo Park, CA, USA) and Illumina MiSeq platforms (Illumina, San Diego, CA, USA). For Illumina DNA sequencing library preparation, gDNA was sheared and size-selected using a bead-based method following the TruSeq DNA Nano kit. For PacBio sequencing, gDNA was sheared with Megaruptor, purified with AMPure PB magnetic beads, and processed using SMRTbell prep kit. A total of 54,575 PacBio HiFi reads (N50 of 11.976 kbp) and 14,789,620 Illumina paired-end raw reads (N50 of 2.233 Mbp) were obtained. Low-quality reads (below 1,000 bp and those in the lowest 10% quality of reads) were removed using Filtlong (v0.2.1) (5). Quality control on the Illumina reads was performed using Fastp (v0.23.4) (6). The PacBio reads were assembled using Trycycler (v0.5.5) (7). Subsets of filtered HiFi reads were subsampled using the “Trycycler subsample” function with a target genome size of 5 Mbps and *de novo* assembled with Raven (v1.8.3) (8), Flye (2.9.4-b1799) (9), and Canu (v1.5) (10). A consensus genome comprising 10 circular contigs was generated from the assemblies using the Trycycler cluster, reconcile, partition, and consensus functions. The consensus contigs were polished with Illumina short reads using Polypolish (v0.6.0) (11) and POLCA within MaSuRCA (v4.0.5) (12). The circularity of the contigs was confirmed twice: once during the Trycycler pipeline assembly and again when mapping the Illumina reads backward. The final genome was annotated with Prokka (v1.14.6) (13), while the publicly available genome was annotated using the NCBI’s Prokaryotic Genome Annotation Pipeline (14–16). Unless otherwise noted, all bioinformatics tools were used with default settings.

The statistics of *P. marcusii* strain LL1 genome are summarized in Table 1. Based on CheckM (v1.2.3) (17), the genome completeness was estimated at 99.39%, with contamination at 1.01%, and contained 3,853 coding sequences (CDS), 9 rRNAs, and 50 tRNAs. Average nucleotide identity (ANI) analysis was performed using the ANI calculator (18), and 97.76% of the DNA sequence was identical to *P. marcusii* strain CP157. The genes involved in the biosynthesis of both PHA (including *phaZ*, involved in PHA depolymerization) and carotenoids (*crtI*, *crtR*, *crtO*, *crtW*, and *crtZ*) were found within the *P. marcusii* LL1 genome. These indicate that *P. marcusii* LL1 is capable of synthesizing PHA and carotenoids, which are compounds with diverse applications.

**TABLE 1:** Final assembly and annotation results showing 10 contigs

Contig name	Length (bp)	GC (%)	Depth (X)	CDS	tRNA	rRNA
Contig1	3,104,276	67.5	122	3023	49	9
Contig2	253,155	68.6	78.5	226	0	0
Contig3	230,595	67.6	95.4	204	1	0
Contig4	187,755	58.6	203	187	0	0
Contig5	159,648	59.7	81.7	151	0	0
Contig6	26,455	60.0	47	22	0	0
Contig7	12,208	61.3	85.5	15	0	0
Contig8	6,659	56.5	69.2	4	0	0
Contig9	11,944	60.5	60.7	12	0	0
Contig10	8,479	51.9	32.2	9	0	0
Total	4,001,174	66.7	119	3853	50	9

## Data Availability

The complete genome sequence of *Paracoccus marcusii* LL1 has been deposited in GenBank under the accession number CP174138 for its chromosome and CP174139-CP174147 for its plasmids; the project data are available under BioProject accession number PRJNA1178628 and BioSample accession number SAMN44473628. The PacBio and Illumina raw sequence reads were deposited in the SRA under the accession numbers SRR31136362 and SRR31732183, respectively.
